# Fréquence cardiaque de récupération et présence de lésions coronaires au cours des cardiopathies ischémiques à l'Institut de cardiologie d'Abidjan en Côte d'Ivoire

**DOI:** 10.48327/mtsi.v3i2.2023.200

**Published:** 2023-05-11

**Authors:** Iklo COULIBALY, Bénédicte BOKA, Hermann YAO, Arnaud EKOU, Gabin TRO, Camille TOURE, Désirée KOUASSI

**Affiliations:** 1 Service Médecine, Institut de cardiologie d'Abidjan, 01 BP V 206 Abidjan, Côte d'Ivoire; 2 Service d'hémodynamique, Institut de cardiologie d'Abidjan, Côte d'Ivoire; 3 Institut de cardiologie de Bouaké, Côte d'Ivoire

**Keywords:** Fréquence cardiaque de récupération, Lésions coronaires, Cardiopathies ischémiques, Épreuve d'effort, Côte d'Ivoire, Afrique subsaharienne, Heart rate recovery, Coronary artery lesions, Ischemic heart disease, Stress test, Côte d'Ivoire, Sub-Saharan Africa

## Abstract

**Introduction:**

La fréquence cardiaque de récupération (FCR) est la différence entre la fréquence cardiaque au maximum de l'effort et celle atteinte à la première minute et/ou à la deuxième minute de la récupération. Cette mesure s'effectue soit au cours d'une récupération passive (arrêt total de l'effort puis patient mis en position assise ou en décubitus dorsal) soit au cours d'une récupération active (pédalage à une charge à 0 watt si bicycle ergométrique ou marche lente si tapis roulant). Elle participe à l’évaluation de la balance vago-sympathique. Une FCR anormale est associée à une mortalité cardiovasculaire connue qui serait corrélée à la présence et la sévérité des coronaropathies. Le but de ce travail était d’évaluer la valeur prédictive de la FCR dans le diagnostic des lésions coronaires dans nos conditions d'exercice.

**Patients et méthode:**

Nous avons mené une étude rétrospective observationnelle de janvier 2010 à février 2020 à l'Institut de cardiologie d'Abidjan, incluant des patients ayant réalisé une coronarographie diagnostique dans les suites d'une épreuve d'effort positive. Les données cliniques, angiographiques et les paramètres d'exercice ont été analysés et comparés chez des patients ayant une FCR anormale et ceux ayant une FCR normale.

**Résultats:**

L’âge moyen de notre échantillon de 41 patients était de 53, 4 ± 9, 6 ans avec une prédominance masculine (sexe-ratio de 3, 6). Chez 8 patients (19, 5%) présentant une anomalie de la FCR, une maladie coronaire était plus souvent présente (p = 0, 02) et celle-ci était plus sévère (p = 0, 003) que chez les patients sans anomalie de la FCR. Ils étaient plus âgés sans significativité statistique (p = 0, 081), et ils avaient une plus faible réserve chronotrope (p = 0, 008) et une fréquence cardiaque maximale plus basse (p = 0, 042). La valeur prédictive positive de la FCR était de 87, 5% et la valeur prédictive négative de 60, 6%.

**Conclusion:**

Dans notre contexte, une FCR anormale est associée à la présence d'une coronaropathie plus sévère. Exploration peu coûteuse, la mesure de la FCR au cours des épreuves d'effort pourrait contribuer au dépistage, à l’évaluation de leur sévérité et au suivi des cardiopathies ischémiques dans nos pays à ressources limitées.

## Abstract (en)

**Introduction:**

Heart rate recovery (HRR) measured during stress tests, assesses the vago-sympathetic balance. It is a known prognostic and predictive parameter of cardiovascular mortality that is believed to be correlated with the presence and severity of coronary artery disease. The aim of this work was to assess the predictive value of heart rate recovery in the diagnostic and severity of coronary lesions in a major metropolis of sub-Saharan Africa where access to specialist care is unevenly distributed.

**Patients and method:**

We conducted a retrospective observational study from January 2010 to February 2020 at the Abidjan Cardiology Institute, including patients who performed a diagnostic coronary angiography after a positive exercise test. Clinical, angiographic and exercise parameters were analyzed and compared in patients with abnormal heart rate recovery (HRR) and those with normal one.

**Results:**

The main study limitation is small sampling due to the cost of the angiographic procedure which limits its realization. We recorded 41 subjects whose mean age was 53.4 ± 9.6 years with a male predominance (sex ratio of 3.6). The predominant age group was between 50 and 60 years. Males were older than females with no significant difference. The predominant cardiovascular risk factors were overweight/obesity (68.29%) and hypertension (61%). Eight patients (19.5%) presenting an abnormal HRR (≤12 bpm) had more significant coronary disease (p=0.02) and more severe ones (p=0.003). Patients with abnormal HRR tended to be older without statistical significance (p=0.081), and had lower chronotropic reserve and maximum heart rate (p=0.008 and p=0.042, respectively). The positive predictive value of HHR was 87.5% and its negative predictive value was 60.6%.

**Conclusion:**

Abnormal HRR can predict the presence of coronary artery disease and its severity. Evaluating HRR during stress tests could help in the detection, evaluation, and monitoring of ischemic heart disease in our resource-limited countries.

## Introduction

La prévalence des cardiopathies ischémiques a fortement progressé dans le monde [13] ainsi qu'en Afrique subsaharienne et particulièrement en Côte d'Ivoire [19, 27]. Elles constituent la première cause de mortalité dans le monde [13]. Si leur diagnostic et leur prise en charge ont bénéficié de l'apport de l'imagerie et des méthodes d'angioplastie, l’épreuve d'effort (EE) reste encore dans nos pays à ressources limitées un moyen d’évaluation d'usage répandu bien que de plus en plus délaissé dans les pays développés [14]. Elle possède cependant un intérêt à la fois diagnostique et pronostique. Parallèlement aux critères diagnostiques habituels, d'autres paramètres ont été proposés pour améliorer la valeur pronostique de l’épreuve d'effort, comme l'analyse cinétique des variations de la fréquence cardiaque (FC) pendant et après l'effort. Il s'agit de l’élévation de la fréquence cardiaque au début de l'effort, de l'incompétence chronotrope et de la fréquence cardiaque de récupération (FCR) après l'effort [2]. Tous ces paramètres sont régulés par le système nerveux autonome, des valeurs anormales étant liées à une diminution de l'effet parasympathique et à une augmentation de l'effet sympathique [2]. L'incompétence chronotrope et la fréquence cardiaque de récupération sont actuellement les paramètres les plus prédictifs. Il a été démontré qu'une décroissance anormale de la FC au cours de la récupération est prédictive d’événements cardiovasculaires majeurs et de mortalité toutes causes confondues chez des individus aussi bien sains que malades [8, 15, 18, 21, 25]. Elle constitue également un facteur de mauvais pronostic chez les coronariens [12] indépendamment de la sévérité des lésions coronariennes [23].

L'objectif de ce travail était d’évaluer la valeur prédictive de la FCR pour le diagnostic et l'estimation de la sévérité des lésions coronaires dans le contexte d'une grande métropole subsaharienne où l'accès aux soins spécialisés est très inégalement réparti.

## Patients Et Méthode

Il s'agissait d'une étude observationnelle, rétrospective à visée analytique. Elle a été menée à l'Institut de cardiologie d'Abidjan (ICA) à partir des registres des services d'hémodynamique et des explorations externes sur des dossiers couvrant une période de 10 ans, de janvier 2010 à février 2020. Parmi toutes les coronarographies réalisées sur la période d’étude, nous avons exploité celles des patients ayant réalisé une épreuve d'effort maximale, positive ou litigieuse. L’épreuve d'effort était positive lorsqu'il existait à la fois des symptômes cliniques (en particulier une douleur thoracique évocatrice) et des anomalies de la repolarisation (sous-décalage du segment ST horizontal ou descendant ≥ 1 mm, 60-80 ms après le point J ou sous-décalage du segment ST ascendant ≥ 1, 5 mm, 80 ms après le point J). L’épreuve d'effort (EE) était considérée comme litigieuse lorsqu'il existait un seul critère (clinique ou électrique).

Ont été exclus les patients:

- ayant déjà une cartographie des coronaires (coronarographie ou Coro scanner);
- souffrant de pathologies pouvant moduler le fonctionnement du système nerveux autonome, notamment les antécédents d'insuffisance cardiaque, d'infarctus du myocarde (IDM) et les accidents vasculaires cérébraux (AVC) [20];
- porteurs de stimulateurs cardiaques ou prenant des médicaments tels que l'adé-nosine, les digitaliques, les inhibiteurs calciques à action chronotrope ou autres anti-arythmiques qui pourraient affecter la fréquence cardiaque de récupération, non interrompus depuis au moins 48 heures [21].

Le recueil des données a été réalisé à l'aide d'une fiche d'enquête standardisée. Les dossiers ont été sélectionnés à partir de la base de données du Registre prospectif des actes en cardiologie interventionnelle (REPACI) du Service d'hémodynamique, puis exploités dans les archives de l'ICA.

Les paramètres étudiés étaient:

- les données épidémiologiques – âge, sexe, données anthropométriques (poids et taille, permettant de calculer l'indice de masse corporelle IMC par la formule Poids (kg)/Taille^2^ (m^2^));
- les facteurs de risque cardiovasculaire;
  - l'hypertension artérielle (HTA), diabète, tabagisme, dyslipidémie, obésité (définie par un IMC ≥ 30 kg/m^2^):
  - l'hypertension artérielle a été définie par une pression artérielle systolique supérieure ou égale à 140 mmHg et/ou une pression artérielle diastolique supérieure ou égale à 90 mmHg, à 3 reprises au cours de deux consultations successives sur une période de 3 mois ou chez les patients sous traitement anti-hypertenseur (mesures hygiéno-diététiques ou médicaments);
  - le diabète a été retenu en présence d'un critère parmi les suivants: hémoglobine glyquée supérieure à 6, 5%, glycémie plasmatique à jeun depuis 8 heures supérieure ou égale à 1, 26 g/l, à 2 reprises, glycémie plasmatique supérieure ou égale à 2 g/l après un test d'hyperglycémie provoquée par voie orale, glycémie plasmatique à n'importe quel moment de la journée > 2 g/l;
  - le tabagisme actif était retenu en cas de tabagisme actuel ou interrompu depuis moins de 3 ans;
  - la dyslipidémie a été définie par un taux de cholestérol total supérieur à 2, 40 g/l et/ou un taux de HDL cholestérol < 0, 40 g/l chez l'homme ou < 0, 50 g/l chez la femme et/ou un taux de LDL cholestérol > 1, 60 g/l;
- le traitement en cours;
- la fraction d’éjection systolique du ventricule gauche (FEVG) calculée au cours de l’échographie doppler cardiaque transthoracique;
- les données de l’épreuve d'effort – critères d'arrêt, fréquence cardiaque de repos (FC repos), tension artérielle de repos (TA repos), fréquence cardiaque maximale (FC max), fréquence cardiaque maximale théorique (FMT), fréquence cardiaque de récupération (FCR).

La FCR est la différence entre la fréquence cardiaque au maximum de l'effort et celle atteinte à la première minute et/ou à la deuxième minute de la récupération. La mesure s'effectue soit au cours d'une récupération passive (arrêt total de l'effort puis patient mis en position assise ou en décubitus dorsal) ou au cours d'une récupération active (pédalage à une charge à 0 watt si bicycle ergométrique ou marche lente si tapis roulant). Elle est anormale lorsqu'elle est ≤ 12 battements par minute (bpm) à la 1^re^ minute ou ≤ 22 bpm à la 2^e^ minute [23]. Nous avons retenu la fréquence cardiaque mesurée à la 1^re^ minute de la récupération active comme préconisé en cas de suspicion d'une coronaropathie [7]. L'EE était considérée comme maximale lorsque la fréquence maximale réelle (FC max) du patient atteint ou dépasse les 85% de sa fréquence cardiaque maximale théorique (FMT) ou par l'apparition de signes d’épuisement ou une incapacité à poursuivre l'exercice [17]. La FMT a été obtenue en utilisant la formule d'Astrand: FMT = 220 – âge; et pour les patients sous bêtabloquants ou autres agents bradycar-disants (lorsqu'ils n'avaient pas été arrêtés depuis au moins 48 heures) la formule suivante: FMT = 164 – 0, 7 × âge [23].

Le bicycle a été l'ergomètre le plus utilisé chez 25 patients (61%).

Les données de la coronarographie (sténose coronaire significative, athérome non sténosant, nombre d'artères sténosées) constituent le standard pour le diagnostic de maladie coronarienne significative dans notre étude et donc pour le calcul des critères de performance de l'altération de la FCR (sensibilité, spécificité, et valeurs prédictives). Les lésions coronaires significatives étaient définies par un rétrécissement de la lumière d'une artère coronaire épicardique (artère interventriculaire antérieure, circonflexe ou coronaire droite) > 70% et > 50% pour le tronc commun. En fonction du nombre de vaisseaux atteints,

- les lésions étaient monotronculaires lorsque la lésion sténosante siégeait sur l'une des artères;
- les lésions étaient bitronculaires lorsque la lésion sténosante siégeait sur deux des artères sus-citées;
- les lésions étaient tritronculaires lorsque les trois troncs étaient atteints.

La saisie des données a été effectuée à l'aide du logiciel statistique EpiData 3.1 puis l'analyse statistique a été réalisée avec le logiciel Stata 12.0.

Les variables quantitatives ont été exprimées sous forme de moyennes ± écart type. Elles ont été comparées par le test de Mann-Whitney/Wilcoxon. Les variables qualitatives ont été exprimées par leurs effectifs et/ou pourcentages. Elles ont été comparées par le test de Chi^2^ ou celui de Fisher lorsque les effectifs théoriques étaient inférieurs à 5. Le seuil de significativité a été retenu pour une valeur de p < 0, 05.

## Résultats

Dans une série de 41 patients, majoritairement masculine (78%) soit un sexe-ratio homme/femme de 3, 6, l’âge moyen était de 53, 4 ± 9, 6 ans avec des extrêmes de 32 et 72 ans et un âge médian de 55 ans. La tranche d’âge prédominante était celle entre 50 et 60 ans. Les hommes étaient plus âgés que les femmes sans différence significative. Le facteur de risque cardiovasculaire prépondérant était l'HTA (61%) (Tableau I).

**Tableau I:** Caractéristiques des patients selon le niveau de la fréquence cardiaque de récupération (ICA, Abidjan, 2010-2020) Patient characteristics by heart rate recovery level (Abidjan Cardiology Institute, 2010-2020)

	FCR normale (> 12 bpm) n = 33	FCR anormale (≤ 12 bpm) n = 8	p
**Facteurs de risque**			
âge (années), m ± ET	52, 2 ± 10,0	58, 8 ± 5,8	0,081
genre masculin	24 (72, 7)	8 (100, 0)	0,164
IMC (kg/m^2^), m ± ET	26, 7 ± 2,7	27, 6 ± 3,4	0,434
HTA	19 (57, 6)	6 (75, 0)	0,448
diabète	13 (39, 4)	2 (25, 0)	0,687
tabagisme actif	3 (9, 1)	2 (25, 0)	0,246
dyslipidémie	20 (60, 6)	3 (37, 5)	0,267
obésité	6 (18, 2)	2 (25, 0)	0,64
**Traitements en cours**			
aspirine	16 (48, 5)	2 (25, 0)	0,429
bêtabloquant	15 (45, 4)	4 (50, 0)	1,000
statine	17 (51, 5)	3 (37, 5)	0,696
**Données angiographiques**			
maladie coronaire significative	13 (39, 4)	7 (87, 5)	0,020
monotronculaire	6 (18, 2)	1 (12, 5)	1,000
bitronculaire	4 (12, 1)	1 12, 5)	1,000
tritronculaire ou TC	3 (9, 1)	5 (62, 5)	0,003

Le test d'effort a été interrompu le plus souvent pour épuisement musculaire (68, 3%). La FCR à la première minute était anormale (≤ 12 bpm) chez 8 patients (19, 5%). Dans 7 cas (87, 5%) on retrouvait une maladie coronaire significative à la coronarographie (p = 0, 02) qui était plus fréquemment tritronculaire ou intéressant le tronc commun (62, 5% contre 9, 1%, p = 0, 003) par comparaison avec les patients ayant une FCR normale.

Les paramètres hémodynamiques de repos n’étaient pas différents dans les deux sous-groupes (FCR normale et anormale). Par contre, après un effort maximal, la fréquence cardiaque au pic de l'effort (p = 0, 042) et la réserve chronotrope (p = 0, 008) étaient plus basses chez les patients avec une FCR anormale (Tableau II).

**Tableau II:** Paramètres retrouvés à l’épreuve d'effort selon la fréquence cardiaque de récupération Parameters found in stress test according to heart rate recovery

	FCR normale (> 12 bpm) n = 33	FCR anormale (≤ 12 bpm) n = 8	p
**Repos**			
PAS (mmHg)	133, 7 ± 13,6	140, 5 ± 12,2	0,268
FC (bpm)	77, 8 ± 12,9	81, 4 ± 11,1	0,472
**Effort maximal**			
PAS (mmHg)	191, 8 ± 11,6	184, 2 ± 12,6	0,265
FC (bpm)	154, 3 ± 12,6	142, 9 ± 18,1	0,042
réserve chronotrope	76, 3 ± 13,5	61, 5 ± 13,8	0,008

Les données sont en moyenne ± écart type FCR: fréquence cardiaque de récupération PAS: pression artérielle systolique FC: fréquence cardiaque

La sensibilité de l'altération de la FCR dans la détection de la maladie coronaire était de 35% et sa spécificité de 95, 2%. La VPP (valeur prédictive positive) de la FCR était de 87, 5% et sa VPN (valeur prédictive négative) de 60, 6% (Tableau III).

**Tableau III:** Évaluation de la performance de la FCR comme méthode prédictive de la détection de la maladie coronaire objectivée par une coronarographie Evaluation of heart rate recovery performance as a predictive method for coronary disease determined by coronary angiography

	Présence de maladie coronaire significative	Absence de maladie coronaire significative	
**FCR anormale**	7	1	VPP = 87, 5%
**FCR normale**	13	20	VPN = 60, 6%
	Se = 35%	Sp = 95, 2%	

FCR: fréquence cardiaque de récupération VPP: valeur prédictive positive VPN: valeur prédictive négative Se: sensibilité Sp: spécificité

## Discussion

Cette étude est la première du genre dans notre contexte géographique. Elle a consisté à mettre en évidence une corrélation entre l'altération de la FCR et la présence de lésions coronaires dans les cardiopathies ischémiques par l'analyse des dossiers de patients suspects de coronaropathie ayant réalisé une coronarographie après une épreuve d'effort positive ou litigieuse. Notre travail a révélé qu'une altération de la FCR à la première minute de récupération dans le post-exercice était associée à la présence de lésions coronaires sévères.

Sur le plan socio-démographique, nous avons retrouvé une prédominance masculine (78%) avec un sexe-ratio de 3, 6 comme dans la majorité des études portant sur la maladie coronaire [10, 13, 18].

La tranche d’âge la plus représentée était celle de 50 à 60 ans avec un âge moyen de 53, 4 ± 9, 6 ans. Si ce résultat se rapprochait de celui observé par Akyüz *et al.* en Turquie [1], il se distinguait cependant de celui observé dans la série américaine de Shelter *et al.* qui retrouvait une population plus âgée [22].

Une FCR anormale à la première minute était significativement associée à la présence de la maladie coronaire [1].

La FCR s'est révélée peu sensible (35%) dans la détection de la maladie coronaire avec cependant une bonne spécificité (95, 2%) comme l'avaient déjà observé Ghaffari [10] et Chaitman [3]. Un tel niveau de spécificité peut conférer à la FCR une utilité dans l’évaluation de la cardiopathie ischémique dans notre contexte, surtout dans le ciblage des patients chez qui un surcroît d'exploration invasive doit être envisagé.

Ce résultat semble cependant controversé puisque Akyüz *et al.* [1] avaient retrouvé plutôt une bonne sensibilité (76, 1%) et une faible spécificité de la FCR dans la détection de la maladie coronaire. Mais la méthode de mesure (récupération en position assise) et donc de seuil de la FCR (21 bpm) dans leur travail pourrait expliquer cette différence. Dans tous les cas, nous pensons qu'un travail ultérieur de plus grande envergure devrait nous permettre d'obtenir des résultats plus probants avec des implications plus précises.

Quant à la sévérité de la maladie coronaire (en particulier les lésions tritronculaires), elle était significativement associée à une mauvaise FCR comme dans de nombreuses études antérieures [4, 5, 6, 9, 10] même si certains travaux [16, 22, 26] n'ont pas retrouvé ce lien. Ils ont cependant démontré que la FCR demeurait un facteur de mauvais pronostic chez les patients coronariens quelle que soit la sévérité de la maladie [22, 26].

Même si les mécanismes par lesquels la maladie coronaire affecte la FCR ne sont pas complètement élucidés, il semble exister un déséquilibre et un dysfonctionnement de la régulation nerveuse autonome appelé neuropathie autonome cardiaque [5, 26]. Elle se manifeste par une altération de la réactivation vagale et un renforcement de l'activité sympathique au maximum de l'effort [24]. Ce phénomène serait responsable de la faible décroissance de la fréquence cardiaque lors de la récupération et d'une altération de la réponse chronotrope [8]. Par ailleurs, une altération de la fréquence cardiaque de récupération serait le témoin d'une dysfonction endothéliale qui fait le lit des lésions athéro-scléreuses telles que les coronaropathies [11].

Notre étude comporte néanmoins des limites. D'une part un très faible échantillonnage du fait du coût de la procédure angiographique qui en limite la réalisation dans notre contexte. Les résultats obtenus montrent certes l'insuffisance de la réduction de la fréquence cardiaque lors de la récupération et la sévérité des lésions coronaires, mais il s'agit d'une étude rétrospective faite chez des patients à probabilité pré-test élevée de maladie coronaire pour lesquels une indication de coronarographie avait été retenue. L'indication de cette dernière pouvait être liée à une forte suspicion clinique de cardiopathie indépendamment des résultats de l’épreuve d'effort. Il serait donc opportun dans un second temps de mettre en évidence ce même lien chez des patients avec une faible probabilité de cardiopathie ischémique avant la coronarographie dans une série prospective.

## Conclusion

L'intérêt pronostique de la fréquence cardiaque de récupération est établi au cours des pathologies cardiovasculaires et particulièrement des cardiopathies ischémiques dans les pays à haut niveau de développement sanitaire. Nos données suggèrent qu'une mauvaise FCR est aussi associée à la présence de la maladie coronaire avec une forte spécificité dans notre contexte ivoirien. Cependant à ce stade, une étude complémentaire avec un plus grand échantillon est nécessaire. Elle permettrait de valider ces résultats et de déterminer la conduite à tenir devant une anomalie de la FCR dans un contexte de suspicion de cardiopathie ischémique dans les pays aux plateaux techniques limités.

## Contribution Des Auteurs

I. Coulibaly: supervision de l’étude, validation des données, prospection bibliographique, relecture, validation du manuscrit

B. Boka: conception de l’étude, prospection bibliographique, supervision de l’étude

H. Yao: analyse des données, interprétation des résultats

A. Ekou: validation du protocole

G. Tro: rédaction du manuscrit

C. Touré: rédaction du manuscrit.

## Liens D'intérêt

Les auteurs ne déclarent aucun lien d'intérêt
